# The effect of alcohol consumption on human physiological and perceptual responses to heat stress: a systematic scoping review

**DOI:** 10.1186/s12940-024-01113-y

**Published:** 2024-09-12

**Authors:** Nathan B. Morris, Nicholas Ravanelli, Georgia K. Chaseling

**Affiliations:** 1 William J. Hybl Sports Medicine and Performance Center, Department of Human Physiology and Nutrition, University of Colorado, Colorado Springs, CO USA; 2 Department of Physiology, Yoo Long Lin School of Medicine, National University of Singapore, Singapore, Singapore; 3 Sydney Nursing School, Faculty of Medicine and Health, The University of Sydney, Sydney, NSW Australia

## Abstract

**Background:**

Ethyl alcohol (ethanol) consumption is ostensibly known to increase the risk of morbidity and mortality during hot weather and heatwaves. However, how alcohol independently alters physiological, perceptual, and behavioral responses to heat stress remains poorly understood. Therefore, we conducted a systematic scoping review to understand how alcohol consumption affects thermoregulatory responses to the heat.

**Methods:**

We searched five databases employing the following eligibility criteria, studies must have: 1) involved the oral consumption of ethanol, 2) employed a randomized or crossover-control study design with a control trial consisting of a volume-matched, non-alcoholic beverage, 3) been conducted in healthy adult humans, 4) reported thermophysiological, perceptual, hydration status markers, and/or behavioral outcomes, 5) been published in English, 6) been conducted in air or water at temperatures of > 28°C, 7) involved passive rest or exercise, and 8) been published before October 4th, 2023.

**Results:**

After removing duplicates, 7256 titles were screened, 29 papers were assessed for eligibility and 8 papers were included in the final review. Across the 8 studies, there were a total of 93 participants (93 male/0 female), the average time of heat exposure was 70 min and average alcohol dose was 0.68 g·kg^1^. There were 23 unique outcome variables analyzed from the studies. The physiological marker most influenced by alcohol was core temperature (lowered with alcohol consumption in 3/4 studies). Additionally, skin blood flow was increased with alcohol consumption in the one study that measured it. Typical markers of dehydration, such as increased urine volume (1/3 studies), mass loss (1/3 studies) and decreased plasma volume (0/2 studies) were not consistently observed in these studies, except for in the study with the highest alcohol dose.

**Conclusion:**

The effect of alcohol consumption on thermoregulatory responses is understudied, and is limited by moderate doses of alcohol consumption, short durations of heat exposure, and only conducted in young-healthy males. Contrary to current heat-health advice, the available literature suggests that alcohol consumption does not seem to impair physiological responses to heat in young healthy males.

**Supplementary Information:**

The online version contains supplementary material available at 10.1186/s12940-024-01113-y.

## Introduction

Each year, there are approximately 20,064 heat-related deaths in North America and 489,075 deaths globally [1], with this number expected to rise by 70% to 100% by the year 2050 [2]. Identifying factors that increase the risk of morbidity and mortality during heatwaves will be critical to help mitigate this tremendous loss of life. One identified risk factor is ethyl alcohol (ethanol) consumption (referred to by its colloquial name “alcohol” for the remainder of this review) [3–9]. Recently, the World Health Organization acknowledged there is no level of safe alcohol consumption for our health [10]. Yet, an estimated 2.3 billion people worldwide consume alcohol [11], and of particular concern, alcohol consumption is seasonal, peaking during summer months [12]. During heatwaves, official heat-health guidelines from major international health authorities such as the Center for Disease Control [13], Red Cross [14], and World Health Organization [15], and national public health authorities such as Drinkaware UK [16] and the American National Weather Service [17], commonly suggest avoiding alcohol use, both for its potential effects on thermoregulation as well its effects as a diuretic. Despite these recommendations, a recent study of the daily habits of 285 participants, from three different countries, found that 15% of the surveyed adults reported alcohol consumption as a “thirst management solution” during heatwaves [18].

There are four main pathways through which alcohol consumption could put a person at risk for heat-related illness: 1) by impairing thermophysiological responses to the heat, 2) by compromising hydration status through its diuretic effect, 3) by impairing behavioral responses, and 4) by impairing decision making. Whether alcohol consumption conclusively affects physiological, perceptual, or behavioral responses to the heat, however, reamins equivocal. For example, all public health authorities recommend against the consumption of alcohol due to its effects on hydrations status [13–15], yet, studies investigating the effect of alcohol on hydration markers have shown hydration status remains unchanged following alcohol consumption, particularly when alcohol is used for rehydration after an athletic event [19, 20]. Similarly, some health guidelines state that alcohol impairs the body’s ability to lose heat during a heatwave [13], however, laboratory studies have reported alcohol consumption increases skin vasodilation, which would help—not hinder—heat loss, as well as reduced core temperatures [21].

Further obfuscating how alcohol may place individuals at greater risk for heat illness is that many public health recommendations concerning alcohol consumption for humans during heat stress have been based on animal studies [22, 23], demonstrating impaired behavioral [24, 25] and physiological [24, 26] responses to the heat. However, given the vast differences in anatomy, metabolism and thermoregulatory mechanisms between humans and animals, the clinical relevance of these findings are limited and caution should be taken when extrapolating these findings to inform human clinical guidelines [27]. Accordingly, the purpose of this systematic scoping review was to search the literature to assess the influence of alcohol consumption on thermal physiological, behavioral, hydrational, and cognitive responses to heat stress in humans.

## Methods

### Search strategy

To identify all available studies investigating the effects of alcohol on thermoregulatory responses, a literature search was performed. Following the creation and organization of the original search terms into a PICO table (Supplementary Table 1), we developed the search strings, using appropriate meSH terms, and translated the searches into the correct format for each database with support from a University of Colorado Colorado Springs Librarian. The systematic search was conducted in Scopus, Academic Search Premier, MEDLINE, CINAHL, and EMBASE and included articles published until October 4th, 2023. The search was conducted using a list of key search terms identified and agreed upon by the authors and organized into a Boolean search strategy (supplementary materials). The review protocol was reported in accordance with the Preferred Reporting Items for Systematic Reviews and Meta Analyses’ guidelines for scoping reviews [28].

### Eligibility criteria

Studies were considered eligible if they: 1) involved the oral consumption of ethanol, 2) employed a randomized or crossover-control study design with a control trial consisting of a volume-matched, non-alcoholic beverage, 3) were conducted in healthy adult humans, 4) reported thermophysiological (core temperature, skin temperature, sweating, and skin blood flow), perceptual (thermal comfort and thermal sensation), hydration status and behavioral outcomes (change in the use of a cooling device, cool seeking behavior, etc.), 5) published in English, 6) conducted in air or water at temperatures of > 28°C, 7) involved passive rest or exercise, and 8) published before October 4th, 2023. In situations where multiple interventions were used (e.g., two different alcohol concentrations), all relevant interventions were included in the data synthesis.

### Study selection and data extraction

Titles and abstracts were screened in duplicate by two different screening teams, consisting of either NM or GC with support from an undergraduate assistant, in order to identify relevant papers using Rayyan title screening software [29]. Following the completion of title screenings, NM and GC compared title inclusion lists and resolved any discrepancies between lists. Data extraction was similarly performed in duplicate, and the following metrics were extracted using a standardized template: first author, year, country of origin, number of participants (male and female), study population, active or passive heat exposure, length of heat exposure, alcohol dose and delivery method, blood alcohol content, ambient temperature and humidity the study was conducted in, the stated aim of the study, the protocol employed by the study, reported outcome measures, main results, discussion points, notes of relevance, and any studies referenced investigating the effects of alcohol on thermoregulation that were not detected in the initial review. Whenever possible, if the stated alcohol dose and blood alcohol content were not in g/kg and g/dL, respectively, standardized dose were calculated based on the reported data. Data extracted from the included papers are presented in tables as means with standard deviation. Subsequently, the extracted data were presented graphically, or in text, as Cohen’s d, with 95% confidence intervals, using standard calculations [30].

## Results

### Search overview

**Fig. 1:**
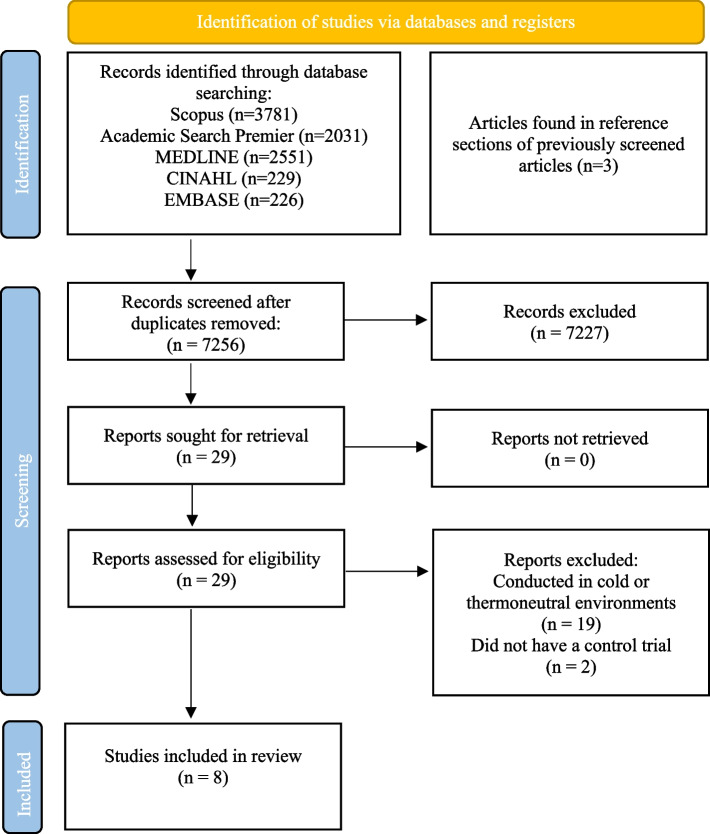
Flow diagram of the review screening process

The screening process of the systemic search is detailed in Fig. 1. Following the removal of duplicate findings between databases (*n* = 7253) and the addition of records identified through other sources (*n* = 3), 7,256 unique titles were screened. From this, 7,227 titles were further excluded. An additional 19 papers were removed as they were conducted below the cut-off threshold of > 28°C and two papers were removed as they did not use a control trial. This left 8 papers that were included in our analysis [31–38].

### Study characteristics

A summary of the study designs for the eight studies included in the review are displayed in Table 1. The year of publication ranged from 1979 to 2015, with only two papers published in the 2000’s. Two of the studies came from from France, while one paper came from Japan, Poland, Spain, Sweden, Canada and the USA, each. Collectively, there were 93 participants tested across the eight papers (an average of 12 participants per study, range: 6 to 27). The primary area of interest was the effect of alcohol on thermoregulatory responses in five papers, markers of hydration status in two of the papers, and cardiovascular responses in one paper.

**Table 1:** Summary of studies investigating the effect of alcohol consumption on thermoregulation, cardiovascular responses, hydration markers, and biomarkers in the heat. Data presented as means with standard deviation, unless otherwise stated. Alcohol dosages have been standardized to grams of alcohol per kilogram of body mass and blood alcohol content has been standardized to grams of alcohol per liter of blood, with original units given in parentheses. BAC blood alcohol content

Article and origin	Study population	Protocol	Dose, Control, Delivery, and Blood Alcohol Content
Passive heat exposure (air)			
Yoda, 2005 [38] Japan	***N*** = 8 (8 M, 0F) **Age:** 26 ± 11 years **Population:** healthy alcohol-tolerant men	90 min exposure to 33 °C and 50%RH air	**Dose:** 0.36 g/kg of ethanol **Delivery:** Oral ingestion **Control:** Volume-matched distilled water **BAC:** Not measured
Livingstone, 1980 [35] Canada	***N*** = 8 (8 M, 0F) **Age:** 25 ± 5 years **Population:** Young healthy Caucasians	90 min passive exposure in 30 °C and 40%RH air, pre-ingestion and 120 min post-ingestion	**Dose:** 50 cc of pure ethanol **Delivery:** Oral ingestion **Control:** 50 cc of orange juice **BAC:** Not measured
Gibiński, 1979 [33] Poland	***N*** = CON: 12 (12 M, 0F) ALC: 15 (15 M, 0 F) **Age:** 29—41 years (range) **Population:** Healthy adults	120 min exposure to 43 °C air	**Dose:** 0.78 g/kg (150 ml of 45% ethanol) **Delivery:** Oral ingestion **Control:** Ad libitum water consumption **BAC:** Not measured
Passive heat exposure (water)			
Allison, 1992 [31] US	***N*** = 6 (6 M, 0F) **Age:** 31 ± 6 years **Population:** healthy adults	40 °C water immersion for 21 min	**Dose:** 0.27 or 0.54 g/kg of 151 proof rum, in 350 ml of caffeine and sugar free cola **Delivery:** Oral ingestion **Control:** 350 ml of caffeine and sugar free cola **BAC:** 0.040 ± 1 0.011 g/dl; 0.077 ± 0.015 g/dl
Mekjavic, 1987 [36] Canada	***N*** = 6 (6 M, 0F) **Age:** Not reported **Population:** Young healthy men	40 °C water immersion for 60 min	**Dose:** 0.79 g/kg (2.5 ml/kg of 40% ethanol) in a 1:2 ratio to orange juice **Delivery:** Oral ingestion **Control:** 7.5 ml/kg of orange juice **BAC:** 0.078 ± 0.01 g/dL (0.078 ± 0.01 g%)
Exercise in hot environments			
Jiménez-Pavón, 2015 [34] Spain	***N*** = 22 (22 M, 0F) **Age**: 21 ± 1 years **Population:** Physically active men	60 min running at 60% VO_2max_ in 35 °C and 60%RH	**Dose:** 0.32 g/kg (660 ml of 4.5% beer) post-exercise followed by ad libitum water consumption **Delivery:** Oral ingestion **Control:** Ad libitum water consumption **BAC:** not measured
Desruelle, 1996 [32] France	***N*** = 8 (6 M, 0F) **Age**: Not reported **Population**: Healthy adults	60 min of exercise at 45% VO_2max_ in 35 °C and 45% RH	**Dose:** 1.20 g/kg of ethanol in a 600 ml cocktail **Delivery:** Oral ingestion **Control:** volume-matched placebo cocktail **BAC:** 0.11 g/dL (SD not reported)
Saini, 1995 [37] France	***N*** = 8 (8 M, 0F) **Age:** 23 ± 3 years **Population:** Healthy men	60 min cycling at 45%VO_2max_ in 35 °C, 35% RH	**Dose:** 1.20 g/kg of ethanol (vodka) mixed with 345 ml of orange juice **Delivery:** Oral ingestion **Control:** 345 ml orange juice with water to match volume **BAC:** 0.108 g/dl (SD not reported) (23.5 mmol/l)

### Study Participants

All 93 of the participants were male (0% female). All eight studies were conducted in young healthy participants. The mean age of the study participants was 27 years, ranging from 21 to 41 years. Age was not reported in two studies, but in both studies the participants were described as “young and healthy males”.

### Study design

**Fig. 2:**
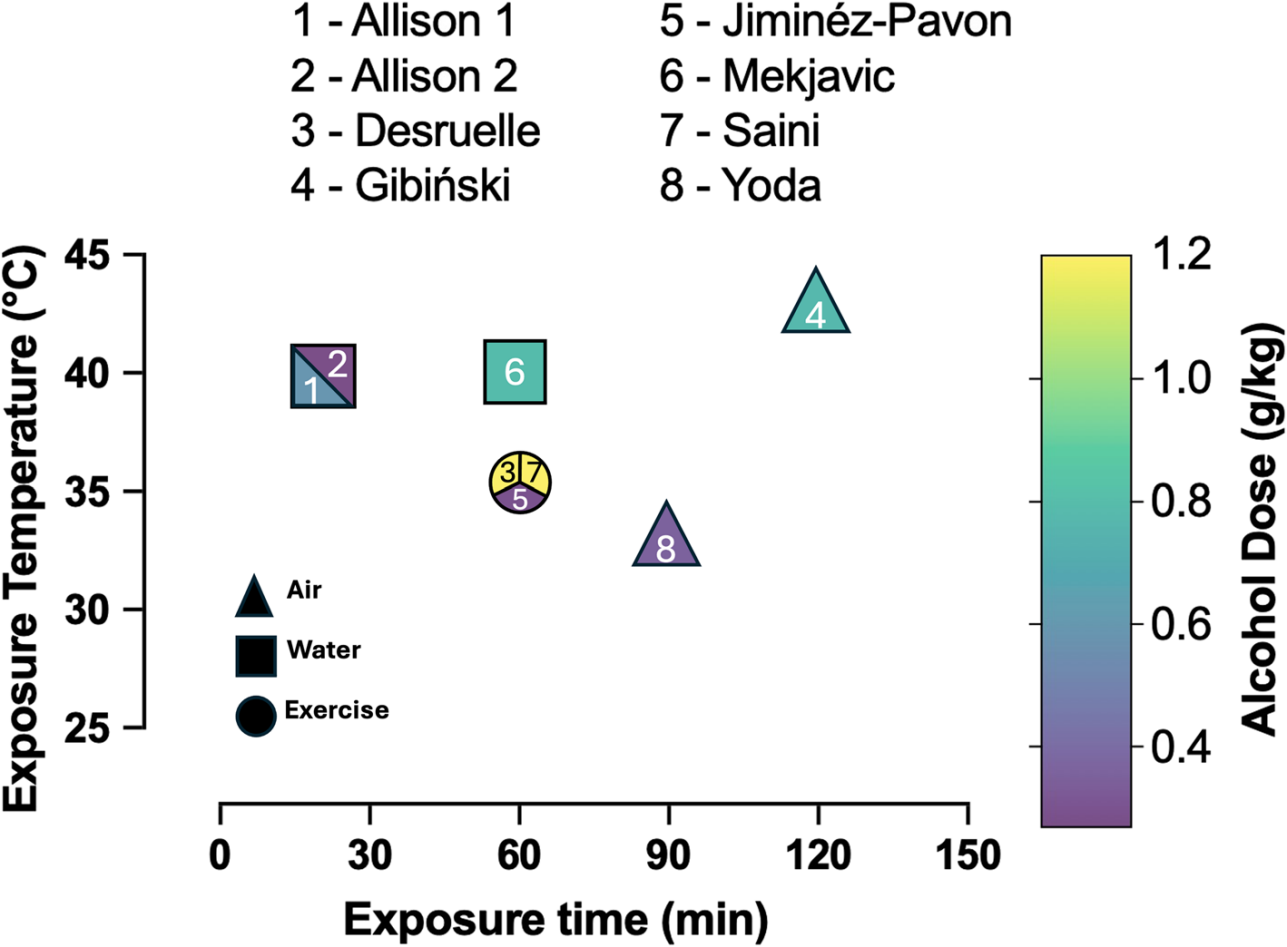
Experimental set up of eight alcohol-standardized studies. One study was not included in this figure because not enough information was provided to calculate a standard dose in g/kg. Studies by number: Allison 1992 (0.54 g/kg dose), 1; Allison 1992 (0.27 g/kg dose), 2; Desruelle 1996, 3; Gibiński 1979, 4; Jiménez-Pavón 2015, 5; Mekjavic 1987, 6; Saini 1995, 7; Yoda 2005, 8

A visual overview of the trial duration, alcohol dose, and heat stress type is depicted in Fig. 2. Three of the papers used active heat stress (i.e. exercise), five papers used passive heat stress only. Of the five passive heat exposures, three were in air temperatures ranging from 30 to 43°C and two were conducted in 40°C water. The average heat stress exposure-time was 70 min, with the shortest duration being 21 min and the longest being 120 min. Between the eight different studies, there were nine different alcohol doses administered. However, in one of the studies [35], neither alcohol dose expressed as g/kg nor the participants’ weights were given, and as such, the dose could not be standardized. Of the eight conditions where the standardized dose could be attained, the average dose was 0.68 g/kg, ranging from 0.27 g/kg to 1.2 g/kg. Of the nine alcohol dose conditions, blood alcohol content was measured in four of them. The average blood alcohol content was 0.082 g/dl, with a range of 0.04 g/dl to 0.11 g/dl.

### Study outcomes

## Thermophysiological markers

**Fig. 3:**
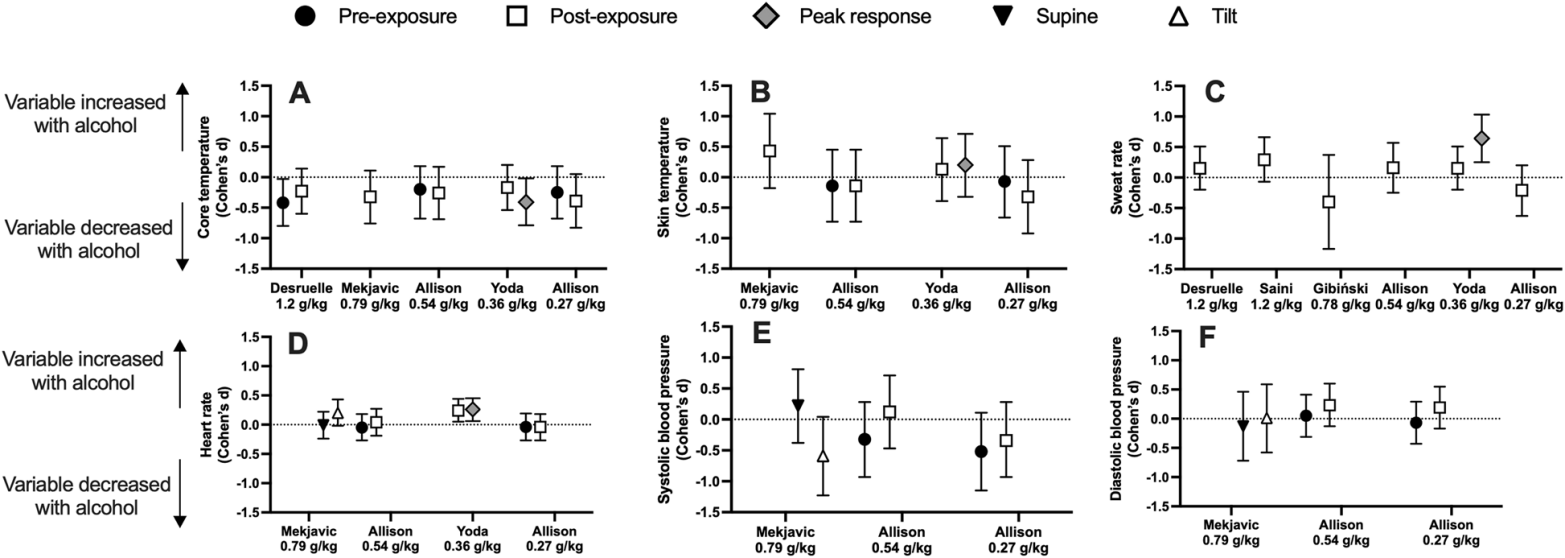
Effect sizes (Cohen’s d) with 95% confidence intervals for core temperature (panel **A**), skin temperature (panel **B**), sweat rate (panel **C**), heart rate (panel **D**), systolic blood pressure (panel **E**) and diastolic blood pressure (panel **F**) when consuming alcohol compared to a volume matched control beverage. Values above the dashed-line denote an increase, whereas below the line denotes a decrease, in the variable with alcohol consumption, relative to control. Closed circles denote physiological responses pre heat exposure, open squares are responses post heat exposure, grey diamonds are the peak response, black downward triangles are during supine positioning following heat exposure and open upward triangles are after a head-up tilt procedure following heat exposure

Individual study data can be found in Table 2 and a visual summary of the thermophysiological effect sizes and 95% confidence intervals can be found in Fig. 3. Core temperature was measured in four studies [31, 32, 36, 38]. Of these studies, one reported no difference in core temperature [31] for either the 0.27g/kg or 0.54 g/kg dosing conditions, two reported a lower resting core temperature with alcohol consumption [32, 38] and two reported a lower end exposure core temperature with alcohol [36, 38], although end exposure core temperature neared statistical significance (*p* = 0.06; d = -0.23[95%CI:-0.60 to 0.14]; *n* = 8) in a third study [32].

**Table 2:** Outcome variables for four studies examining the effects of alcohol consumption on thermoregulatory and cardiovascular responses to the heat

		Desruelle 1.20 g/kg			Mekjavic 0.79 g/kg			Allison 0.54 g/kg			Yoda^a^ 0.32 g/kg			Allison 0.27 g/kg		
		CON	ALC	p	CON	ALC	p	CON	ALC	p	CON	ALC	p	CON	ALC	p
Tre (°C)	SE	37.38 ± 0.28	37.04 ± 0.37	< 0.05				37.22 ± 0.23	37.12 ± 0.21	> 0.75				37.22 ± 0.23	37.13 ± 0.42	> 0.75
	EE	38.21 ± 0.23	38.00 ± 0.45	0.06	38.28 ± 0.22	37.98 ± 0.47	NR	37.65 ± 0.25	37.5 ± 0.11	> 0.75				37.65 ± 0.25	37.50 ± 0.11	> 0.75
Tint (°C)	SE										37.37 ± 0.25	37.11 ± 0.27	< 0.01			
	EE										37.32 ± 0.18	37.21 ± 0.32	< 0.01			
Tes (°C)	SE							36.75 ± 0.21	36.62 ± 0.21	> 0.81				36.75 ± 0.21	36.63 ± 0.27	> 0.81
	EE							37.77 ± 0.18	37.58 ± 0.21	> 0.81				37.77 ± 0.18	37.65 ± 0.19	> 0.81
Tsk (°C)	SE	"Not affected by alcohol" No values reported						33.40 ± 0.97	33.3 ± 0.6	> 0.28	0.06 ± 0.56	0.26 ± 0.70	> 0.05	33.40 ± 0.97	33.2 ± 0.75	> 0.28
	EE				30.7 ± 2.45	32.1 ± 0.24	NR	35.43 ± 1.05	34.8 ± 1.31	> 0.28	0.13 ± 0.71	0.28 ± 0.72	> 0.05	35.43 ± 1.05	34.8 ± 1.31	> 0.28
SBF (ml⋅min^−1^⋅100 g^−1^)	SE										0.62 ± 2.16	2.41 ± 3.86	< 0.05			
	EE										0.15 ± 1.13	0.41 ± 0.97	> 0.05			
Sweat Rate (mg⋅min^−1^⋅cm^−2^)	SE	0.04 ± 0.03	0.04 ± 0.03	> 0.05				0.22 ± 0.27	0.13 ± 0.19	> 0.51	0.06 ± 0.09	0.26 ± 0.15	< 0.01	0.22 ± 0.27	0.01 ± 0.12	> 0.51
	EE	1.1 ± 0.31	1.28 ± 0.51	> 0.05				4.14 ± 0.98	4.83 ± 2.09	> 0.51	0.02 ± 0.13	0.07 ± 0.08	< 0.01	4.14 ± 0.98	3.57 ± 1.05	> 0.51
Heart Rate (bpm)	SE				77 ± 7	76 ± 11	> 0.05	76 ± 17	73 ± 12	> 0.75	-2 ± 4	8 ± 11	< 0.01	76 ± 17	73 ± 8	> 0.75
	EE				97 ± 7	109 ± 16	> 0.05	108 ± 12	106 ± 8	> 0.75	0 ± 6	12 ± 12	< 0.05	108 ± 12	110 ± 9	> 0.75
Thermal Sensation (AU)	SE										0.08 ± 0.43	0.86 ± 1.60	< 0.05			
	EE										0.06 ± 0.52	0.06 ± 0.27	> 0.05			
Thermal Comfort (AU)	SE							1.5 ± NR	1.5 ± NR	> 0.45	-0.42 ± 0.61	0.69 ± 0.86	< 0.05	1.5	1.5	> 0.45
	EE							0.5 ± NR	0.5 ± NR	> 0.45	-0.24 ± 0.63	0.00 ± 0.42	> 0.05	0.5	0.5	> 0.45
SBP (mmHg)	S				114 ± 21	120 ± 21	> 0.05	116 ± 9	108 ± 10	> 0.28				116 ± 9	111 ± 11	> 0.28
	HT				117 ± 8	106 ± 13	> 0.05	113 ± 17	105 ± 10	> 0.28				113 ± 17	116 ± 10	> 0.28
DBP (mmHg)	S				60 ± 14	57 ± 15	> 0.05	72 ± 10	70 ± 9	> 0.12				72 ± 10	73 ± 6	> 0.12
	HT				71 ± 18	71 ± 10	> 0.05	55 ± 10	60 ± 9	> 0.12				55 ± 10	61 ± 9	> 0.12
SV (ml)	S				86 ± 19	84 ± 27	> 0.05									
	HT				63 ± 10	61 ± 16	> 0.05									

*SE* Start exposure, *EE* End exposure, *S* Supine, *HT* post head-tilt procedure, *Tre* rectal temperature, *Tint* Intestinal temperature, *Tes* esophageal temperature, *Tsk* skin temperature, *SBF* Skin blood flow, *SBP* Systolic blood pressure, *DBP* Diastolic blood pressure, *SV* stroke volume, *NR* Not reported. ^a^For all Yoda values, peak value was put in place of start exposure, as exposure began immediately following alcohol ingestion

One study measured whole-body sweat rate, reporting no difference with alcohol consumption compared to a control fluid [33]. Four studies measured local sweat rate [31–33, 38], two studies reported no difference [31, 33], one study reported a non-significant increase with alcohol consumption (reported *p* value: > 0.05; calculated effect size: 0.15 [95%CI: -0.20 to 0.51]; *n* = 8) [32], and one study reported significantly elevated local sweat rates with alcohol ingestion [38].

One study measured skin blood flow [38] that found a significant increase with alcohol ingestion. Four studies measured skin temperature [31, 32, 35, 38] and none of them found any differences. Additionally, one study [36] inferred a reduction in total peripheral resistance (i.e. greater skin blood flow) with alcohol ingestion based on a lower diastolic blood pressure, with an elevated heart rate. In total, three studies measured heart rate [31, 36, 38], one showing no difference [31], one showing a significant elevation in heart rate [38] with alcohol consumption, and one reported a non-significant elevation in heart rate with alcohol consumption (*p* > 0.05; d = 0.20 [95%CI: -0.02 to 0.43]; *n* = 6) [36]. Of the two studies measuring blood pressure [31, 36], one found a reduction in mean arterial pressure with alcohol ingestion [36], while the other found no difference [31].

Thermal sensation and thermal comfort were only measured in two studies [31, 38], with conflicting results. In the first study [31], both thermal sensation and thermal comfort were unaffected by alcohol and insufficient data were presented to calculate effect sizes. In the other study [38], thermal sensation was greater (participants felt hotter) with alcohol consumption (end exposure: d = 0.73 [95%CI: 0.04 to 1.42]; peak response: d = 0.46 [95%CI: -0.18 to 1.10]), however, they felt more comfortable in the heat following alcohol consumption compared to a control fluid (end exposure: d = 0.36 [95%CI: -0.27 to 0.99]; peak response: d = 1.17 [95%CI: 0.37 to 1.96]).

## Hydration and biomarkers of fluid regulation

**Fig. 4:**
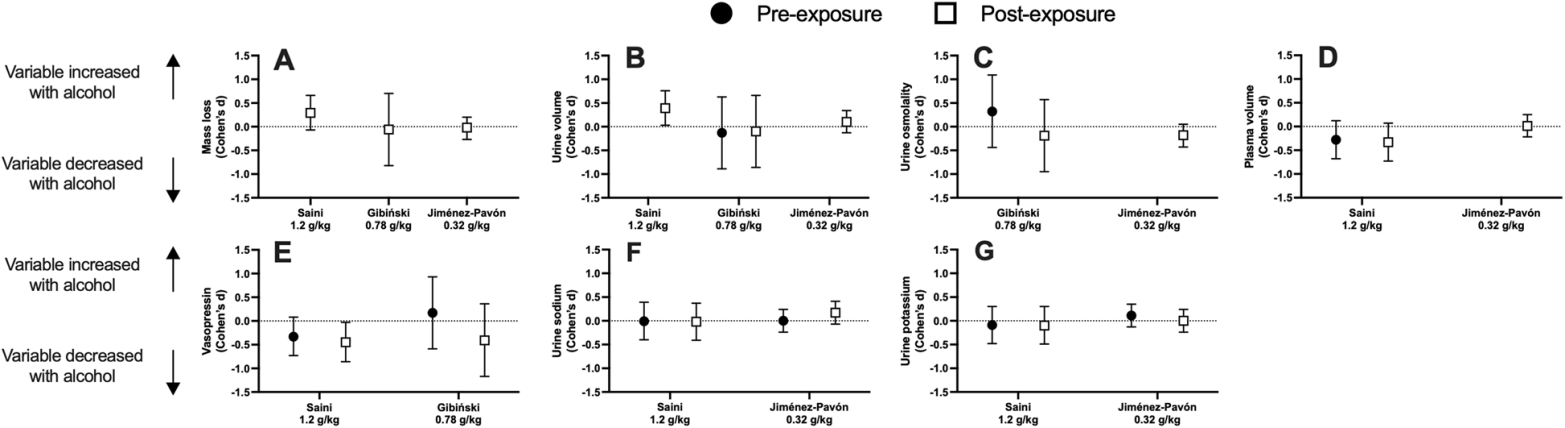
Effect sizes (Cohen’s d) with 95% confidence intervals for mass loss (panel **A**), urine volume (panel **B**), urine osmolality (panel **C**), plasma volume (panel **D**), vasopressin (panel **E**), urione sodium (panel **E**), and urine potassium (panel **G**) when consuming alcohol compared to a volume matched control beverage. Values above the dashed-line denote an increase, whereas below the line denotes a decrease, in the variable with alcohol consumption, relative to control. Closed circles denote physiological responses pre heat exposure and open squares denote responses post heat exposure

A detailed summary of the hydration marker and biomarker data can be found in Table 3 and a visual summary of the effect sizes with 95%CI can be found in Fig. 4. Three studies examined whole-body mass loss [33, 34, 37] and one found greater mass losses with alcohol consumption [37]. Three studies investigated the effect of alcohol consumption on urine volume [33, 34, 37]. Of these studies one [37] found that urine output was higher with alcohol consumption, while the others found no differences. Two studies [33, 34] measured urine osmolality and found no differences with alcohol consumption compared to a control fluid.

**Table 3:** Outcome variables for three studies examining the effects of alcohol consumption on hydration and hormone markers of fluid regulation

		Saini (1.2 g/kg)			Gibiński (0.78 g/kg)			Jiménez-Pavón (0.32 g/kg)		
		Control	Alcohol	p	Control	Alcohol	p	Control	Alcohol	p
Mass loss (kg)	SE							74.2 ± 6.5	74.3 ± 6.8	= 0.23
	EE	0.84 ± 0.11	0.96 ± 0.18	< 0.05	1.54 ± 0.29	1.53 ± 0.21	> 0.05	72.4 ± 6.3	72.6 ± 6.7	
Urine volume (g)	SE				161 ± 124	146 ± 112	> 0.05			
	EE	278 ± 163	480 ± 229	< 0.05	52 ± 17	50 ± 22	> 0.05	223 ± 245	281 ± 245	= 0.70
Natriuretic peptide (pg⋅ml^−1^)	SE	14.74 ± 4.78	13.28 ± 5.21	> 0.05						
	EE	21.57 ± 5.86	18.12 ± 6.51	> 0.05						
Aldosterone (pg⋅ml^−1^)	SE	266 ± 126	148 ± 101	> 0.05						
	EE	677 ± 232	631 ± 241	> 0.05						
Vasopressin (pg⋅ml^−1^)	SE	0.76 ± 0.10	0.56 ± 0.26	< 0.05	3.9 ± 5.2	4.7 ± 4.3	> 0.05			
	EE	1.31 ± 0.52	0.78 ± 0.61	< 0.05	16.2 ± 14.7	10.0 ± 15.7	> 0.05			
Plasma osmolality (mosm⋅kg^−1^)	SE	280 ± 6	297 ± 6	> 0.05						
	EE	281 ± 4	301 ± 4	> 0.05						
Plasma Potassium (mmol⋅L^−1^)	SE	4.19 ± 0.14	4.15 ± 0.24	> 0.001				4.6 ± 0.4	4.7 ± 0.4	= 0.28
	EE	4.72 ± 0.17	4.67 ± 0.28	> 0.001				4.7 ± 0.4	4.7 ± 0.3	
Plasma Sodium (mmol⋅L^−1^)	SE	138 ± 1	138 ± 8	> 0.05				138 ± 2	138 ± 2	= 0.95
	EE	140 ± 12	140 ± 2	> 0.05				138 ± 2	139 ± 3	
Plasma volume (Δ%)	SE	-1.27 ± 3.17	-3.25 ± 2.93	NR						
	EE	-5.10 ± 2.56	-7.51 ± 3.66	NR				-5.3 ± 8.3	-5.1 ± -5.7	= 0.44
Urine osmolality (mosm)	SE				719 ± 299	802 ± 215	> 0.05			
	EE				855 ± 192	818 ± 191	> 0.05	681.50 ± 181.04	587.17 ± 252.23	= 0.28
Sweat loss (mg)	EE				2.878 ± 1.047	2.493 ± 0.894	> 0.05			
Hematocrit (%)	SE							45.7 ± 3.0	45.1 ± 2.9	= 0.45
	EE							46.6 ± 2.5	45.6 ± 2.2	= 0.45

*SE* Start exposure, *EE* End exposure, *NR* Not reported

Two studies measured plasma volume [34, 37], with both studies finding no difference with alcohol consumption. Two studies looked at circulating antidiuretic hormone/vasopressin [33, 37]. One study found lower vasopressin levels with alcohol consumption [37], while the other found no differences. Two studies examined the effect of alcohol on plasma sodium and potassium concentrations [34, 37], with no differences in either plasma sodium or potassium levels with alcohol consumption. One study [37] examined the effect of alcohol consumption on plasma osmolality (pre exposure: d = 1.55 [95%CI: 0.97 to 2.13]; end exposure: d = 2.36 [95%CI: 1.60 to 3.13]), finding a very large increase with alcohol consumption. Additionally, this study [37] examined natriuretic peptide (pre exposure: d = -0.13 [95%CI: -0.52 to 0.27]; end exposure: d = -0.24 [95%CI: -0.64 to 0.15]) and aldosterone (pre exposure: d = -0.45 [95%CI: -0.86 to -0.04]; end exposure: d = -0.09 [95%CI: -0.48 to 0.31]), finding no differences in either. Finally, one study [34] examined the effect of alcohol on hematocrit (pre exposure: d = -0.09 [95%CI: -0.33 to 0.15]; end exposure: d = -0.42 [95%CI: -0.42 to 0.05]), and found no effect of alcohol consumption.

## Discussion

This review sought to to determine what evidence currently exists demonstrating the impact of alcohol consumption on human physiological and perceptual responses to heat exposure. The most consistent finding was a lowered core temperature with alcohol consumption (observed in 3/4 studies). Skin blood flow was higher with alcohol consumption in the one study that measured it, and local sweat rate was higher with alcohol consumption in one of the four studies that measured it. Moreover, traditional hydration markers were generally unaltered with alcohol consumption compared to a control beverage, with the exception of increased urine volume and reduced antidiruretic hormone and body mass in one study. Collectively, these limited findings suggest that acute alcohol consumption generally does not impair physiological responses during heat stress.

### The effect of alcohol on physiological responses

Findings from this review demonstrate that alcohol consumption increased skin blood flow in the one study that measured it and reduced core temperature in three of the four studies where it was measured. These findings are consistent with research dateing back to 1861 that demonstrated the vasodilatory effect of alcohol consumption [39, 40]. This peripheral vasodilation following alcohol consumption likely leads to a redistribution of warm blood from the viscera to the extremities, thereby leading to a lower resting core temperature.

In addition to lowering core temperature by redistributing blood to the skin, this process can alter thermal perception and influence thermal sensation and behavior [41]. Only two studies in this review examined alcohol's effects on thermal comfort: one found no difference, while the other reported improved comfort, despite increased feelings of warmth [38]. No studies explored the effects of alcohol consumption on thermoregulatory behavior. The increased risk of hospitalization during heatwaves with alcohol consumption could be due to reduced thermal discomfort, potentially leading to inadequate cooling behaviors. More research is needed, especially on heat-vulnerable populations like the elderly, who already have reduced thermal perception [42]. Additionally, while alcohol is known to affect cognition [42–45], its interaction with heat stress remains unexplored.

This review found limited evidence to support the notion that alcohol ostensibly leads to dehydration and should be avoided during heatwaves [13–17], with only the study using the highest alcohol dose (1.2 g/kg) demonstrating increased markers for dehydration (e.g. greater urine output and mass loss, and lower plasma vasopressin, with alcohol consumption) [37]. These findings are consistent with other studies examining the effect of low doses of alcohol consumption on hydration markers in thermoneutral conditions, where low doses of alcohol did not affect hydration status [19, 20], but higher doses delayed rehydration post exercise [19]. Further, an increase in urine volume of 100 ml per 10 g of ethanol ingested, independently of the fluid volume consumed, has been previously reported [46]. Moreover, the average heat stress exposure-time of the analyzed studies was 93 min, the shortest being 21 min and the longest being 310 min (5.2 h). In contrast to these short study durations, increases in morbidity and mortality during a heatwave typically increase following the third day of elevated tempratures [47]. Therefore, whether the dehydrating effects of alcohol, especially at higher doses, begin to compond over multiple days, crtitically needs to be investigated.

### Limitations of the reviewed articles

The largest omission observed in this review was the complete lack of female participants in any of the included studies. This absence of female representation is particularly concerning considering the known discrepancy between how alcohol affects women compared to men [48]. Similarly, there was a lack of ethnic diversity among study populations. Seven studies included in this review were from primarily Caucasian countries whereas only one [38] was conducted in a non-Caucasian country (Japan). This lack of multi ethnic group representation is important due to the well-known differences in alcohol metabolism between Asians (particularly Japanese, Chinese, and Koreans) and Caucasians [49–51]. Specifically, these Asian populations have a genotypical lack of aldehyde dehydrogenase, the enzyme responsible for metabolizing acetaldehyde, thereby causing the skin flush reaction [52]. This may be an important consideration given the one study on Japanese participants observed the strongest physiological responses to alcohol, such as a prolonged reduction in core temperature, increases in skin blood flow, sweating, and heart rate, and greater feelings of comfort, despite feeling warmer [38].

In general, the sample size of the studies was low with an average of 12 participants per study (range: 6—27 participants per study). In several of these studies, the authors reported consistent but not statistically significant differences in physiological responses with alcohol consumption compared to a control fluid. As such, the lack of differences observed in some of these studies may be due to an inadequate sample size. Further, the average age of the participants studied was 25 years (range: 21—41 years). As lab-based physiological studies have established that thermoregulatory function can decrease past the age of 40 years [53], and that age-related increases in morbidity and mortality during heatwaves typically occurs in those above the age of 65 years [54], future studies should examine alcohol consumption in older populations, to determine whether the effects of alcohol on physiological responses is age-dependent.

As mentioned above, the dose and peak blood alcohol content used and observed in the analyzed studies was relatively low compared to recreational alcohol doses. Specifically, the average blood alcohol content from the analyzed studies was 0.082 g/dl, with a range of 0.04 g/dl to 0.11 g/dl. This is equivalent to just over the legal driving limit of 0.08 g/dl in most American states [55], Canada, Singapore, and most African countries [56]. Conversely, alcohol levels in epidemiological and case studies demonstrating an association between temperature and increased morbidity and mortality, typically report objectively higher BAC values than this, such as 0.2 g/dl [3] and 0.22 g/dl [9], or non-numerical categorizations, suggesting higher levels of alcohol consumption, such as “alcohol misuse” [4], “alcohol misuse disorder” [6], “alcoholic dementia” [3], and “alcohol abuse disorder” [5]. Future studies should consider higher doses of alcohol to better understand the effects of alcohol consumed at higher concentrations. These low doses may also explain why, in this review, few markers of hydration status were affected by alcohol consumption, as previous studies have found that the diuretic effect of alcohol typically occurs at higher levels of alcohol consumption [37, 57].

All studies focused on the effects of acute alcohol consumption on physiological and perceptual responses in healthy young individuals, without considering long-term alcohol use and abuse. Approximately 10% of Americans qualify as having alcohol use disorder [58], as well as 1.4% of the global population [59], which is well known to cause damage to the body’s vasculature [60], chronically increase blood pressure [60–63], and inhibit the endothelia from producing nitric oxide, thereby greatly impeding peripheral vasodilation [64, 65]. As such, heat exposure in this population would likely result in a diminished thermoregulatory vasodilation response, putting this group at greater risk for heat stress and heat related illness. As such, future research of how chronic alcohol users/abusers respond to heat stress are needed.

A final consideration for future studies on the effect of alcohol consumption on thermoregulation is how alcohol may interact with other diseases and medications. In one study comparing occlusive artery disease patients to young healthy controls, alcohol consumption increased cutaneous vasodilation in the young-healthy but not the occlusive artery patients [66]. Accordingly, alcohol consumption could be particularly dangerous for any health condition in which the peripheral vasculature is impaired. Similarly, for those taking anti-hypertensive medications, such as alpha-blockers, alcohol can interact with the anti-hypertensives to cause hypotension [67]. Combined with the lowering of blood pressure caused by heat exposure [68], this could greatly the increase the risk of dizziness, loss of consciousness, and falls during heatwaves [69].

## Conclusions

Findings from the limited studies included in this review demonstrate that acute alcohol consumption does not negatively influence thermoregulation or hydration and hormone markers of fluid balance in the heat, compared to a control fluid. Despite these findings epidemiological research still demonstrates a well-established association between alcohol consumption and a greater morbidity and mortality risk during heatwaves. Therefore, research in this area will be crucial to understand the impact of alcohol on overall health outcomes during heat exposure, including potential interactions with pre-existing conditions, long-term alcohol use, and the effects on behavior and cognition.

## Declerations

All authors and those named in the acknowledgements consented to the publication of this manuscript. All review data are published within the manuscript and supplementary file. None of the authors have any competing interests to disclose. Georgia Chaseling was supported by the SOLVE-CHD Australian Government National Health and Medical Research Council (NHMRC) Synergy Grant (Grant no:1182301). NBM and GKC designed the review and conducted the title screening and data extraction. NBM, GKC, and NR contributed to the writing and editing of the manuscript, as well as the production of the figures. The authors would like to thank Susan Vandagriff for her help preparing the search strings for the review, as well as Anna Carrier and Taigan Lowman for their help screening titles.

## Supplementary Information

Supplementary Material 1.

## Data Availability

No datasets were generated or analysed during the current study.
