# Correction: The effect of alcohol consumption on human physiological and perceptual responses to heat stress: a systematic scoping review

**DOI:** 10.1186/s12940-024-01129-4

**Published:** 2024-10-15

**Authors:** Nathan B. Morris, Nicholas Ravanelli, Georgia K. Chaseling

**Affiliations:** 1 William J. Hybl Sports Medicine and Performance Center, Department of Human Physiology and Nutrition, University of Colorado, Colorado Springs, CO USA; 2 Department of Physiology, Yoo Long Lin School of Medicine, National University of Singapore, Singapore, Singapore; 3 Sydney Nursing School, Faculty of Medicine and Health, The University of Sydney, Sydney, NSW Australia

**Correction: Environ Health 23, 73 (2024)**

10.1186/s12940-024-01113-y

Following publication of the original article [1], the author reported that in Table 1, the Study Population seems to be merged with the next column.

**Table:** 

Incorrect		Correct
Study Population	Study Population	
Passive heat exposure (air)	Passive heat exposure (air)	
**N** = 8 (8M, 0F)		**N** = 8 (8M, 0F)
**Age**: 26 ± 11 years		**Age**: 26 ± 11 years
**Population**: healthy alcohol-tolerant men		**Population**: healthy alcohol-tolerant men
**N** = 8 (8M, 0F)		**N** = 8 (8M, 0F)
**Age**: 25 ± 5 years		**Age**: 25 ± 5 years
**Population**: Young healthy Caucasians		**Population**: Young healthy Caucasians
**N** = CON: 12 (12 M, 0F)		**N** = CON: 12 (12 M, 0F)
ALC: 15 (15 M, 0 F)		ALC: 15 (15 M, 0 F)
**Age**: 29–41 years (range)		**Age**: 29–41 years (range)
**Population**: Healthy adults		**Population**: Healthy adults
Passive heat exposure (water)		Passive heat exposure (water)
**N** = 6 (6M, 0F)		**N** = 6 (6M, 0F)
**Age**: 31 ± 6 years		**Age**: 31 ± 6 years
**Population**: healthy adults		**Population**: healthy adults
**N** = 6 (6M, 0F)		**N** = 6 (6M, 0F)
**Age**: Not reported		**Age**: Not reported
**Population**: Young healthy men		**Population**: Young healthy men
Exercise in hot environments		
**N** = 22 (22M, 0F)		**N** = 22 (22M, 0F)
**Age**: 21 ± 1 years		**Age**: 21 ± 1 years
**Population**: Physically active men		**Population**: Physically active men
**N** = 8 (6M, 0F) **Age**: Not reported **Population**: Healthy adults		60 min of exercise at 45% VO_2max_ in 35°C and 45% RH
**N** = 8 (8M, 0F) **Age**: 23 ± 3 years **Population**: Healthy men		60 min cycling at 45%VO_2max_ in 35°C, 35% RH

The original article has been updated.
