# Supplementary material for: The effect of alcohol consumption on human physiological and perceptual responses to heat stress: a systematic scoping review
Source: Environ Health. 2024 Sep 12;23:73. doi: 10.1186/s12940-024-01113-y (PMC11391823; doi:10.1186/s12940-024-01113-y)
Supplement: Supplementary file 1 — Supplementary Material 1. [file 12940_2024_1113_MOESM1_ESM.docx]

**Supplementary Materials.**

| **Stressors/setting** | **Intervention** | **Outcome variables** |
| --- | --- | --- |
| Heat stress  Heatwave  Heat wave  Heat  Extreme heat  Exercise  Physical activity | Alcohol  Ethanol  Beer  Wine  Vodka  Spirits  Whiskey | Core temperature  Rectal temperature  Skin temperature  Oesophageal temperature  Tympanic temperature  Physiology  Physiological response  Sweat  Sweat losses  Sweating  Skin blood flow  Behaviour  Heart rate  Blood pressure  Thermal comfort  Thermal discomfort  Thermal sensation  Dehydration  Hydration |

Search terms used in systematic search of the literature.

**Supplementary materials – search terms for databases**

**Medline**

((((((((heat stress) OR (heatwave)) OR (heat wave)) OR (heat)) OR (extreme heat)) OR (exercise)) OR (physical activity)) AND (((((((alcohol) OR (ethanol)) OR (beer)) OR (wine)) OR (vodka)) OR (spirits)) OR (whiskey))) AND (((((((((((((((((((core temperature) OR (rectal temperature)) OR (skin temperature)) OR (oesophageal temperature)) OR (tympanic temperature)) OR (physiology)) OR (physiological response)) OR (sweat)) OR (sweat losses)) OR (sweating)) OR (skin blood flow)) OR (behavior)) OR (heart rate)) OR (blood pressure)) OR (thermal comfort)) OR (thermal discomfort)) OR (thermal sensation)) OR (dehydration)) OR (hydration))

**CINAHL and Academic Search Premier**

((MH "Exercise Physiology") OR (MH "Heat Stress Disorders+") OR (MH "Heat-Shock Response") OR extreme heat OR hot temperature* OR Heat OR (Heat N5 (shock OR stress OR illness OR syncope* OR fatigue OR cramp OR exhaustion OR prostration OR collapse OR stroke)) OR Heatstroke OR (exertional heat N5 (illness* OR stress*))) AND ((MH "Alcohol Drinking") OR alcoholic beverage* OR Alcohol OR Ethanol OR Alcohol Drinking) AND ((MH “Body Temperature Regulation”+) OR (MH “Body Temperature”+) OR (MH “Stress, Physiological”) OR (MH “Homeostasis”+) OR (MH “Behavior”) OR (MH “Heart Rate”) OR (MH "Blood Pressure”+) OR (MH “Dehydration”) OR (heat n3 (adaptation OR tolerance OR loss)) OR Body Temperature Regulation OR thermoregula* OR ((skin OR body OR organ OR core OR rectal OR oesophageal OR tympanic) N5 temperature* ) OR ((physiological OR abiotic OR abiotic OR metabolic OR biotic OR biological OR water) N5 stress N5 (response OR react*)) OR Homeostasis OR Autoregulation OR sweat* OR behavio* OR acceptance process* OR ((heart OR cardiac OR pulse) N5 rate) OR heartbeat* OR cardiac chronotrop* OR ((diastolic OR pulse OR systolic) N5 pressure) OR thermosensing* OR temperature sense OR Dehydration OR skin blood flow OR (thermal N5 (comfort OR discomfort OR sensation)) OR Hydration)

**Scopus Search**

( ( TITLE-ABS-KEY ( ( ( skin OR body OR organ OR core OR rectal OR oesophageal OR tympanic ) W/5 temperature* ) OR ( body AND temperature AND regulation ) OR ( thermoregulat* ) OR ( temperature AND sense ) OR ( thermosens* ) OR ( thermal W/5 ( comfort OR discomfort OR sensation ) ) OR ( heat W/5 ( adaptation OR tolerance OR loss ) ) OR ( homeostasis ) OR ( acceptance AND process ) OR ( autoregulate* ) OR ( sweat* ) OR ( ( heart OR cardiac OR pulse ) W/5 rate ) OR heartbeat* OR cardiac AND chronotrop* OR ( ( diastolic OR pulse OR systolic ) W/5 pressure ) OR ( behavio* ) OR ( ( physiological OR abiotic OR abiotic OR metabolic OR biotic OR biological OR water ) W/5 ( rate OR response OR react* ) ) OR ( physiological W/5 stress ) OR ( blood AND pressure ) OR ( skin AND blood AND flow ) OR ( *hydration ) ) ) OR ( INDEXTERMS ( ( "Thermotolerance" ) OR ( "Body Temperature Regulation" ) OR ( "Body Temperature" ) OR ( "stress, physiological" ) OR ( "Homeostasis" ) OR ( "Behavior" ) OR ( "Heart Rate" ) OR ( "Blood Pressure" ) OR ( "Thermosensing" ) OR ( "Dehydration" ) ) ) ) AND ( ( TITLE-ABS-KEY ( ( ( alcohol* OR ethanol ) W/5 ( beverage* OR drinking ) ) OR alcohol OR ethanol ) ) OR ( INDEXTERMS ( ( "Alcoholic Beverages" ) OR ( "Alcohol Drinking" ) ) ) ) AND ( ( TITLE-ABS-KEY ( ( heat W/5 ( stress OR shock OR stroke OR exhaustion OR prostration OR collapse OR illness OR syncope OR fatigue OR cramp ) ) OR ( hot AND temperature* ) OR ( heat ) OR ( extreme AND heat ) OR ( exertional AND heat ) OR ( cold W/5 ( shock OR stress OR injur* OR temperature* ) ) OR ( cold ) OR ( extreme AND cold ) OR ( exercise AND physiology ) OR ( physical AND activity AND physiology ) OR ( physical AND exercise AND physiology ) ) ) OR ( INDEXTERMS ( ( "Heat Stress Disorders" ) OR ( "Heat-Shock Response" ) OR ( "Hot Temperature" ) OR ( "Extreme Heat" ) OR ( "Cold-Shock Response" ) OR ( "Extreme Cold Weather" ) OR ( "Cold Temperature" ) OR ( "Heat Exhaustion" ) OR ( "Heat Stroke" ) OR ( "Exercise/physiology" ) ) ) )
